# *N*′-[6-(3,5-Dimethyl-1*H*-pyrazol-1-yl)-1,2,4,5-tetra­zin-3-yl]butano­hydrazide

**DOI:** 10.1107/S1600536811048033

**Published:** 2011-11-16

**Authors:** Qi-Dong Yan, Feng Xu, Jun Xu

**Affiliations:** a Department of Biological &Chemical Engineering, Taizhou Vocational & Technical college, Taizhou, 318000, People’s Republic of China

## Abstract

In the title compound, C_11_H_16_N_8_O, the tetra­zine and pyrazole rings form a dihedral angle of 48.75 (2)°. In the crystal, N—H⋯O and N—H⋯N hydrogen bonds link the mol­ecules into layers parallel to (101).

## Related literature

For related structures, see: Xu *et al.* (2010 ▶, 2011 ▶). For applications of 1,2,4,5-tetra­zine derivatives, see: Sauer (1996 ▶).
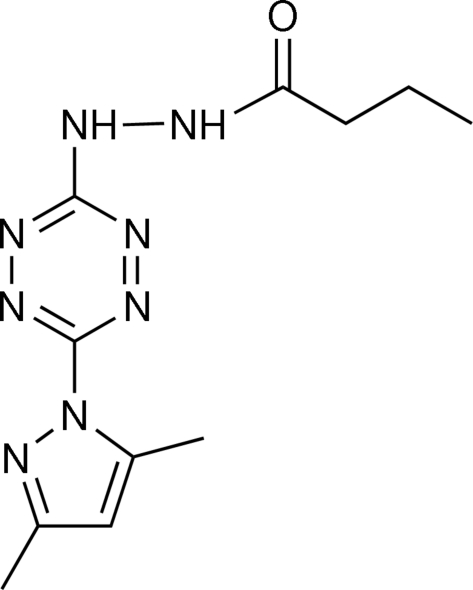


## Experimental

### 

#### Crystal data

C_11_H_16_N_8_O
*M* *_r_* = 276.32
Monoclinic, 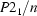

*a* = 10.977 (3) Å
*b* = 7.688 (2) Å
*c* = 15.887 (5) Å
β = 99.798 (5)°
*V* = 1321.2 (6) Å^3^
*Z* = 4
Mo *K*α radiation
μ = 0.10 mm^−1^
*T* = 103 K
0.40 × 0.37 × 0.33 mm

#### Data collection

Rigaku AFC10/Saturn724+ diffractometer
11624 measured reflections
3019 independent reflections
2570 reflections with *I* > 2σ(*I*)
*R*_int_ = 0.025

#### Refinement

*R*[*F* ^2^ > 2σ(*F* ^2^)] = 0.036
*wR*(*F* ^2^) = 0.098
*S* = 1.00
3019 reflections
192 parameters
H atoms treated by a mixture of independent and constrained refinement
Δρ_max_ = 0.30 e Å^−3^
Δρ_min_ = −0.22 e Å^−3^

### 

Data collection: *CrystalClear* (Rigaku/MSC, 2008 ▶); cell refinement: *CrystalClear*; data reduction: *CrystalClear*; program(s) used to solve structure: *SHELXS97* (Sheldrick, 2008 ▶); program(s) used to refine structure: *SHELXL97* (Sheldrick, 2008 ▶); molecular graphics: *PLATON* (Spek, 2009 ▶); software used to prepare material for publication: *SHELXL97*.

## Supplementary Material

Crystal structure: contains datablock(s) global, I. DOI: 10.1107/S1600536811048033/cv5192sup1.cif

Structure factors: contains datablock(s) I. DOI: 10.1107/S1600536811048033/cv5192Isup2.hkl

Supplementary material file. DOI: 10.1107/S1600536811048033/cv5192Isup3.cml

Additional supplementary materials:  crystallographic information; 3D view; checkCIF report

## Figures and Tables

**Table 1:** Hydrogen-bond geometry (Å, °)

*D*—H⋯*A*	*D*—H	H⋯*A*	*D*⋯*A*	*D*—H⋯*A*
N14—H14*N*⋯O17^i^	0.903 (15)	1.923 (16)	2.8221 (15)	173.8 (14)
N15—H15*N*⋯N8^ii^	0.880 (16)	2.008 (16)	2.8851 (16)	174.5 (15)

Symmetry codes: (i) 
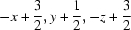
; (ii) 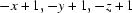.

**Fig. 1.**
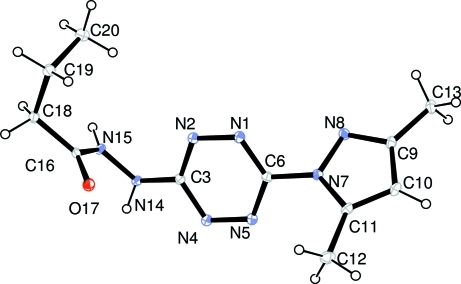
The molecular structure of (I) shown with 30% probability displacement ellipsoids.

**Fig. 2.**
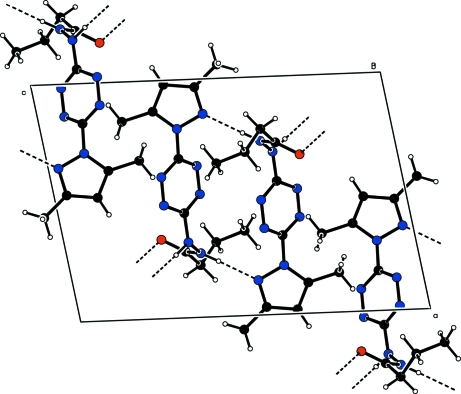
A portion of the crystal packing viewed down the b axis. N—H···O and N—H···N hydrogen bonds are shown as dashed lines.

## supplementary crystallographic information

### Comment

1,2,4,5-Tetrazine derivatives exhibit a wide spectrum of antiviral and antitumor properties. They have been used in pesticides and herbicides (Sauer, 1996). In continuation of our search for the structure-activity relationships of 1,2,4,5-tetrazine derivatives (Xu *et al.*, 2010; 2011), we present here the title compound (I).

In (I) (Fig.1), the tetrazine and pyrazole rings form a dihedral angle of 48.75 (2)°. The N14/N15/C16/O17 and C16/C18/C19 planes make the dihedral angles of 82.56 (2)° and 83.83 (2)°, respectively, with the tetrazine ring. Intermolecular N—H—N and N—H—O hydrogen bonds (Table 1) link molecules into layers parallel to (101) plane (Fig. 2).

### Experimental

3,6-Bis(3,5-dimethyl-1*H*-pyrazol-1-yl)-1,2,4,5-tetrazine (3.0 mmol), chloroform (10 ml) and pyridine(0.25 ml,3.1 mmol) were mixed. Butyryl chloride(3.0 mmol) in chloroform (10 ml) was added dropwise with stirring at room temperature. After the starting 1,2,4,5-tetrazine was completely consumed (the reaction courses was monitored by TLC, ethyl acetate system), evaporation of the chloroform, crude product was obtained and purified by preparative thin-layer chromatography over silica gel GF254(2 mm) (dichloromethane: petroleum ether=1:1). The solution of the compound in anhydrous ethanol was concentrated gradually at room temperature to afford single crystals, which was suitable for X-ray diffraction.

### Refinement

N-bound H atoms were located on a difference map and isotropically refined with N—H bond length restrained to 0.89 (2) Å. Methyl H atoms were placed in calculated positions with C—H = 0.96 Å and torsion angles were refined to fit the electron density, with *U*_iso_(H) = 1.5*U*_eq_(C). Other C-bound H atoms were placed in calculated positions with C—H = 0.93 Å, and refined in riding mode, with *U*_iso_(H) = 1.2*U*_eq_(C).

### Crystal data

**Table:**

C_11_H_16_N_8_O	*F*(000) = 584
*M**_r_* = 276.32	*D*_x_ = 1.389 Mg m^−^^3^
Monoclinic, *P*2_1_/*n*	Mo *K*α radiation, λ = 0.71073 Å
Hall symbol: -P 2yn	Cell parameters from 3486 reflections
*a* = 10.977 (3) Å	θ = 3.3–27.5°
*b* = 7.688 (2) Å	µ = 0.10 mm^−^^1^
*c* = 15.887 (5) Å	*T* = 103 K
β = 99.798 (5)°	Block, red
*V* = 1321.2 (6) Å^3^	0.40 × 0.37 × 0.33 mm
*Z* = 4	

### Data collection

**Table:**

Rigaku AFC10/Saturn724+ diffractometer	2570 reflections with *I* > 2σ(*I*)
Radiation source: Rotating Anode	*R*_int_ = 0.025
graphite	θ_max_ = 27.5°, θ_min_ = 3.3°
Detector resolution: 28.5714 pixels mm^-1^	*h* = −13→14
phi and ω scans	*k* = −9→9
11624 measured reflections	*l* = −20→20
3019 independent reflections	

### Refinement

**Table:**

Refinement on *F*^2^	Primary atom site location: structure-invariant direct methods
Least-squares matrix: full	Secondary atom site location: difference Fourier map
*R*[*F*^2^ > 2σ(*F*^2^)] = 0.036	Hydrogen site location: inferred from neighbouring sites
*wR*(*F*^2^) = 0.098	H atoms treated by a mixture of independent and constrained refinement
*S* = 1.00	*w* = 1/[σ^2^(*F*_o_^2^) + (0.0569*P*)^2^ + 0.316*P*] where *P* = (*F*_o_^2^ + 2*F*_c_^2^)/3
3019 reflections	(Δ/σ)_max_ = 0.001
192 parameters	Δρ_max_ = 0.30 e Å^−^^3^
0 restraints	Δρ_min_ = −0.22 e Å^−^^3^

### Special details

**Table:**

Geometry. All e.s.d.'s (except the e.s.d. in the dihedral angle between two l.s. planes) are estimated using the full covariance matrix. The cell e.s.d.'s are taken into account individually in the estimation of e.s.d.'s in distances, angles and torsion angles; correlations between e.s.d.'s in cell parameters are only used when they are defined by crystal symmetry. An approximate (isotropic) treatment of cell e.s.d.'s is used for estimating e.s.d.'s involving l.s. planes.
Refinement. Refinement of *F*^2^ against ALL reflections. The weighted *R*-factor *wR* and goodness of fit *S* are based on *F*^2^, conventional *R*-factors *R* are based on *F*, with *F* set to zero for negative *F*^2^. The threshold expression of *F*^2^ > σ(*F*^2^) is used only for calculating *R*-factors(gt) *etc*. and is not relevant to the choice of reflections for refinement. *R*-factors based on *F*^2^ are statistically about twice as large as those based on *F*, and *R*- factors based on ALL data will be even larger.

### Fractional atomic coordinates and isotropic or equivalent isotropic displacement parameters (Å^2^)

**Table:**

	*x*	*y*	*z*	*U*_iso_*/*U*_eq_	
O17	0.66813 (8)	0.18530 (11)	0.72170 (5)	0.0179 (2)	
N1	0.36379 (9)	0.45388 (13)	0.57707 (6)	0.0166 (2)	
N2	0.48309 (9)	0.41717 (13)	0.58523 (6)	0.0165 (2)	
N4	0.52508 (9)	0.63680 (13)	0.69398 (6)	0.0178 (2)	
N5	0.40619 (9)	0.67242 (13)	0.68429 (6)	0.0178 (2)	
N7	0.20565 (9)	0.62907 (13)	0.61117 (6)	0.0153 (2)	
N8	0.14658 (9)	0.65755 (13)	0.52903 (6)	0.0159 (2)	
N14	0.68389 (9)	0.49646 (13)	0.64665 (6)	0.0149 (2)	
N15	0.73094 (9)	0.34414 (13)	0.61788 (6)	0.0150 (2)	
C3	0.56031 (11)	0.51505 (15)	0.64082 (7)	0.0142 (2)	
C6	0.33149 (11)	0.58299 (15)	0.62452 (7)	0.0147 (2)	
C9	0.03300 (11)	0.70598 (15)	0.53650 (8)	0.0167 (2)	
C10	0.01821 (11)	0.70610 (16)	0.62297 (8)	0.0195 (3)	
H10	−0.0548	0.7345	0.6447	0.023*	
C11	0.12946 (11)	0.65738 (15)	0.66942 (8)	0.0175 (3)	
C12	0.16684 (13)	0.62803 (19)	0.76288 (8)	0.0249 (3)	
H12A	0.0950	0.6437	0.7913	0.030*	
H12B	0.2313	0.7116	0.7860	0.030*	
H12C	0.1988	0.5094	0.7729	0.030*	
C13	−0.05815 (11)	0.75347 (17)	0.45905 (8)	0.0205 (3)	
H13A	−0.0631	0.8804	0.4538	0.025*	
H13B	−0.1396	0.7069	0.4643	0.025*	
H13C	−0.0316	0.7043	0.4082	0.025*	
C16	0.72224 (10)	0.19424 (15)	0.65988 (7)	0.0146 (2)	
C18	0.78094 (11)	0.03842 (15)	0.62567 (7)	0.0170 (3)	
H18A	0.8220	−0.0332	0.6741	0.020*	
H18B	0.8451	0.0783	0.5932	0.020*	
C19	0.68652 (12)	−0.07353 (16)	0.56757 (8)	0.0192 (3)	
H19A	0.7237	−0.1886	0.5601	0.023*	
H19B	0.6138	−0.0922	0.5957	0.023*	
C20	0.64377 (12)	0.00742 (17)	0.48013 (8)	0.0217 (3)	
H20A	0.6048	0.1201	0.4869	0.026*	
H20B	0.5839	−0.0699	0.4458	0.026*	
H20C	0.7151	0.0242	0.4513	0.026*	
H14N	0.7349 (14)	0.550 (2)	0.6894 (10)	0.028 (4)*	
H15N	0.7670 (15)	0.351 (2)	0.5726 (10)	0.028 (4)*	

### Atomic displacement parameters (Å^2^)

**Table:**

	*U*^11^	*U*^22^	*U*^33^	*U*^12^	*U*^13^	*U*^23^
O17	0.0175 (4)	0.0219 (4)	0.0153 (4)	0.0035 (3)	0.0055 (3)	0.0029 (3)
N1	0.0149 (5)	0.0183 (5)	0.0168 (5)	0.0010 (4)	0.0032 (4)	−0.0008 (4)
N2	0.0146 (5)	0.0180 (5)	0.0169 (5)	0.0016 (4)	0.0023 (4)	−0.0015 (4)
N4	0.0161 (5)	0.0196 (5)	0.0175 (5)	0.0025 (4)	0.0019 (4)	−0.0026 (4)
N5	0.0160 (5)	0.0198 (5)	0.0174 (5)	0.0027 (4)	0.0021 (4)	−0.0013 (4)
N7	0.0152 (5)	0.0187 (5)	0.0125 (5)	0.0019 (4)	0.0037 (4)	0.0006 (4)
N8	0.0146 (5)	0.0191 (5)	0.0141 (5)	0.0013 (4)	0.0025 (4)	0.0015 (4)
N14	0.0146 (5)	0.0152 (5)	0.0150 (5)	0.0011 (4)	0.0024 (4)	−0.0027 (4)
N15	0.0163 (5)	0.0157 (5)	0.0142 (5)	0.0021 (4)	0.0058 (4)	−0.0011 (4)
C3	0.0168 (6)	0.0138 (5)	0.0121 (5)	0.0009 (4)	0.0029 (4)	0.0028 (4)
C6	0.0158 (5)	0.0153 (6)	0.0133 (5)	0.0009 (4)	0.0034 (4)	0.0019 (4)
C9	0.0142 (5)	0.0149 (6)	0.0216 (6)	0.0001 (4)	0.0048 (5)	0.0000 (4)
C10	0.0178 (6)	0.0200 (6)	0.0228 (6)	0.0028 (5)	0.0092 (5)	−0.0002 (5)
C11	0.0200 (6)	0.0166 (6)	0.0180 (6)	0.0006 (5)	0.0088 (5)	−0.0009 (5)
C12	0.0294 (7)	0.0297 (7)	0.0175 (6)	0.0039 (6)	0.0096 (5)	0.0015 (5)
C13	0.0145 (6)	0.0218 (6)	0.0246 (6)	0.0018 (5)	0.0017 (5)	0.0007 (5)
C16	0.0113 (5)	0.0186 (6)	0.0131 (5)	0.0012 (4)	−0.0001 (4)	0.0005 (4)
C18	0.0168 (6)	0.0182 (6)	0.0163 (5)	0.0036 (5)	0.0037 (4)	0.0012 (5)
C19	0.0216 (6)	0.0164 (6)	0.0199 (6)	0.0004 (5)	0.0048 (5)	0.0000 (5)
C20	0.0216 (6)	0.0240 (7)	0.0189 (6)	−0.0028 (5)	0.0023 (5)	−0.0015 (5)

### Geometric parameters (Å, °)

**Table:**

O17—C16	1.2332 (14)	C10—H10	0.9500
N1—N2	1.3243 (14)	C11—C12	1.4887 (17)
N1—C6	1.3304 (15)	C12—H12A	0.9800
N2—C3	1.3453 (16)	C12—H12B	0.9800
N4—N5	1.3166 (14)	C12—H12C	0.9800
N4—C3	1.3602 (15)	C13—H13A	0.9800
N5—C6	1.3349 (15)	C13—H13B	0.9800
N7—C11	1.3663 (15)	C13—H13C	0.9800
N7—N8	1.3723 (13)	C16—C18	1.5049 (16)
N7—C6	1.4068 (15)	C18—C19	1.5301 (17)
N8—C9	1.3258 (15)	C18—H18A	0.9900
N14—C3	1.3515 (15)	C18—H18B	0.9900
N14—N15	1.3883 (14)	C19—C20	1.5216 (17)
N14—H14N	0.904 (16)	C19—H19A	0.9900
N15—C16	1.3432 (15)	C19—H19B	0.9900
N15—H15N	0.880 (17)	C20—H20A	0.9800
C9—C10	1.4104 (17)	C20—H20B	0.9800
C9—C13	1.4932 (17)	C20—H20C	0.9800
C10—C11	1.3674 (17)		
			
N2—N1—C6	117.26 (10)	H12A—C12—H12B	109.5
N1—N2—C3	116.57 (10)	C11—C12—H12C	109.5
N5—N4—C3	116.84 (10)	H12A—C12—H12C	109.5
N4—N5—C6	116.92 (10)	H12B—C12—H12C	109.5
C11—N7—N8	111.97 (10)	C9—C13—H13A	109.5
C11—N7—C6	129.54 (10)	C9—C13—H13B	109.5
N8—N7—C6	118.45 (9)	H13A—C13—H13B	109.5
C9—N8—N7	105.01 (9)	C9—C13—H13C	109.5
C3—N14—N15	119.47 (10)	H13A—C13—H13C	109.5
C3—N14—H14N	119.3 (10)	H13B—C13—H13C	109.5
N15—N14—H14N	114.6 (10)	O17—C16—N15	121.87 (11)
C16—N15—N14	119.89 (10)	O17—C16—C18	122.49 (11)
C16—N15—H15N	122.5 (10)	N15—C16—C18	115.63 (10)
N14—N15—H15N	117.6 (10)	C16—C18—C19	112.20 (10)
N2—C3—N14	119.92 (10)	C16—C18—H18A	109.2
N2—C3—N4	125.35 (11)	C19—C18—H18A	109.2
N14—C3—N4	114.73 (10)	C16—C18—H18B	109.2
N1—C6—N5	126.63 (11)	C19—C18—H18B	109.2
N1—C6—N7	116.88 (10)	H18A—C18—H18B	107.9
N5—C6—N7	116.49 (10)	C20—C19—C18	113.12 (10)
N8—C9—C10	110.69 (11)	C20—C19—H19A	109.0
N8—C9—C13	120.23 (10)	C18—C19—H19A	109.0
C10—C9—C13	129.07 (11)	C20—C19—H19B	109.0
C11—C10—C9	106.57 (11)	C18—C19—H19B	109.0
C11—C10—H10	126.7	H19A—C19—H19B	107.8
C9—C10—H10	126.7	C19—C20—H20A	109.5
N7—C11—C10	105.75 (10)	C19—C20—H20B	109.5
N7—C11—C12	123.70 (11)	H20A—C20—H20B	109.5
C10—C11—C12	130.47 (11)	C19—C20—H20C	109.5
C11—C12—H12A	109.5	H20A—C20—H20C	109.5
C11—C12—H12B	109.5	H20B—C20—H20C	109.5
			
C6—N1—N2—C3	−0.22 (15)	C11—N7—C6—N5	−46.50 (17)
C3—N4—N5—C6	1.40 (16)	N8—N7—C6—N5	130.92 (11)
C11—N7—N8—C9	0.71 (13)	N7—N8—C9—C10	−0.93 (13)
C6—N7—N8—C9	−177.14 (10)	N7—N8—C9—C13	178.23 (10)
C3—N14—N15—C16	−68.57 (14)	N8—C9—C10—C11	0.84 (14)
N1—N2—C3—N14	−173.71 (10)	C13—C9—C10—C11	−178.22 (12)
N1—N2—C3—N4	6.04 (17)	N8—N7—C11—C10	−0.20 (14)
N15—N14—C3—N2	−20.85 (16)	C6—N7—C11—C10	177.35 (11)
N15—N14—C3—N4	159.37 (10)	N8—N7—C11—C12	176.86 (11)
N5—N4—C3—N2	−6.68 (17)	C6—N7—C11—C12	−5.59 (19)
N5—N4—C3—N14	173.08 (10)	C9—C10—C11—N7	−0.36 (13)
N2—N1—C6—N5	−5.02 (18)	C9—C10—C11—C12	−177.15 (13)
N2—N1—C6—N7	175.72 (10)	N14—N15—C16—O17	3.44 (17)
N4—N5—C6—N1	4.37 (18)	N14—N15—C16—C18	−177.48 (9)
N4—N5—C6—N7	−176.37 (10)	O17—C16—C18—C19	80.97 (14)
C11—N7—C6—N1	132.83 (12)	N15—C16—C18—C19	−98.10 (12)
N8—N7—C6—N1	−49.75 (15)	C16—C18—C19—C20	74.42 (13)

### Hydrogen-bond geometry (Å, °)

**Table:**

*D*—H···*A*	*D*—H	H···*A*	*D*···*A*	*D*—H···*A*
N14—H14N···O17^i^	0.903 (15)	1.923 (16)	2.8221 (15)	173.8 (14)
N15—H15N···N8^ii^	0.880 (16)	2.008 (16)	2.8851 (16)	174.5 (15)

Symmetry codes: (i) −*x*+3/2, *y*+1/2, −*z*+3/2; (ii) −*x*+1, −*y*+1, −*z*+1.
